# [^203/212^Pb]Pb-VMT-α-NET as a novel theranostic agent for targeted alpha radiotherapy—first clinical experience

**DOI:** 10.1007/s00259-025-07269-0

**Published:** 2025-04-09

**Authors:** Enrico Michler, David Kästner, Marc Pretze, Holger Hartmann, Robert Freudenberg, Michael K. Schultz, Ralph A. Bundschuh, Jörg Kotzerke, Claudia Brogsitter

**Affiliations:** 1 Department of Nuclear Medicine, University Hospital Carl Gustav Carus, Technische Universität Dresden, Fetscherstraße 74, 01307 Dresden, Germany; 2 Perspective Therapeutics Inc., Coralville, IA USA

**Keywords:** ^203^Pb, ^212^Pb, VMT-α-NET, Neuroendocrine tumors, Theranostic, Targeted alpha therapy

## Abstract

**Purpose:**

^203/212^Pb is a promising theranostic isotope pair for targeted alpha therapy (TAT) of neuroendocrine tumors (NET). VMT-α-NET is a novel SSTR2 targeting peptide that can be labeled with both isotopes. The aim of this work was to perform first clinical investigations of [^203/212^Pb]Pb-VMT-α-NET regarding imaging, biokinetics, tolerability and response.

**Methods:**

12 patients (9 m/3 w; mean age 71, range 60–84) with progressive metastatic GEP-NET grade 1–3 received diagnostic imaging with [^203^Pb]Pb-VMT-α-NET (4.9 MBq/kg bw) up to 24 h p.i. (whole body & SPECT/CT) and, if eligible, a single dose of [^212^Pb]Pb-VMT-α-NET therapy (1.2 MBq/kg bw) after exhaustion of all current therapies (including [^177^Lu]Lu- & [^225^Ac]Ac-DOTATATE), and post-treatment imaging with [^212^Pb]Pb-VMT-α-NET up to 24 h p.i. (whole body & SPECT/CT). Clinical and laboratory parameters were monitored. A visual and quantitative comparison was made with [^68^ Ga]Ga-DOTATATE PET scans before and 3 months after therapy.

**Results:**

No high-grade adverse effects were observed in all patients evaluated with [^203^Pb]Pb-VMT-α-NET. All patients showed an initial high, but lesion-dependent heterogeneous intratumoral accumulation, comparable to [^68^ Ga]Ga-DOTATATE PET. Treatment with [^212^Pb]Pb-VMT-α-NET was also well tolerated by all patients without high-grade or serious adverse side effects. Post-therapeutic PET scans and tumor marker controls showed stable findings in all patients up to 3 months after treatment.

**Conclusion:**

Imaging with [^203^Pb]Pb-VMT-α-NET followed by a single dose of [^212^Pb]Pb-VMT-α-NET appears to be well tolerated with promising efficacy, even in a heterogenous and heavily pretreated patient population. Further studies are warranted to examine tolerability and efficacy over multiple treatment cycles in larger patient populations.

## Introduction

Nuclear medicine plays a critical role in the management of neuroendocrine tumors (NETs), which are rare and diverse neoplasms arising from neuroendocrine cells distributed throughout the body, primarily in the gastrointestinal tract. The therapeutic landscape for NETs has evolved significantly with the advancement of peptide receptor radionuclide therapy (PRRT). This molecularly targeted therapy employs the use of radiolabeled somatostatin analogs to deliver lethal doses of radiation directly to tumor cells that overexpress somatostatin receptors, such as the somatostatin subtype 2 receptor (SSTR2) [1]. The most commonly used radiopharmaceutical used in PRRT is [^177^Lu]Lu-DOTATATE, which outperformed long-acting octreotide administrations in a prospective phase three trial and was therefore approved by the European Medicines Agency and US Food and Drug Administration in 2017 and 2018, respectively [2]. However, with an objective tumor response rate of only 18% for [^177^Lu]Lu-DOTATATE, there is still much room for improvement. In recent years, targeted alpha therapy (TAT) has emerged as a promising approach to address this problem. Alpha particles can cause substantial DNA damage due to their high linear energy transfer, resulting in effective tumor control with minimal exposure to surrounding tissues. This therapy is particularly promising for patients with SSTR2-overexpressing cancers that are refractory to β-PRRT.

**Fig. 1:**
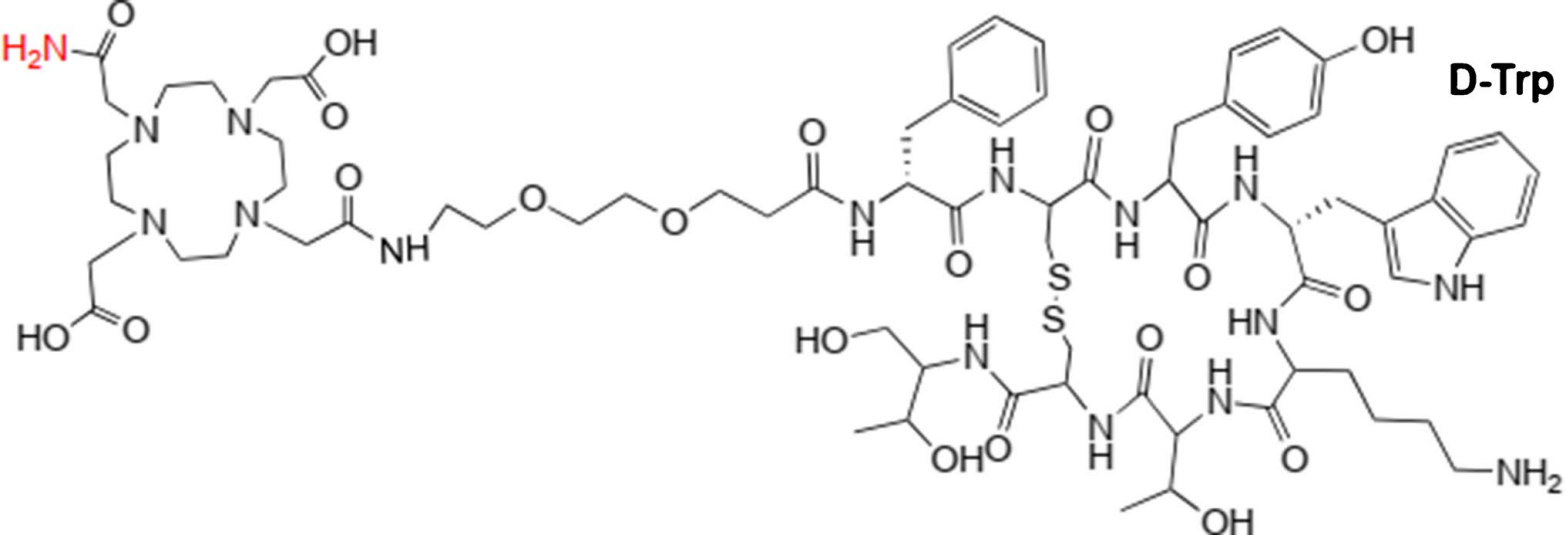
Structure of VMT-α-NET (a PSC-TOC derivative)

A novel development in the field of TAT for NETs is the use of the theranostic pair [^203^Pb]Pb-VMT-α-NET and [^212^Pb]Pb-VMT-α-NET (Fig. 1). The gamma ray emitter ^203^Pb serves as a single-photon emission computed tomography (SPECT) imaging agent with relatively flexible imaging time points due to its half-life of 51.9 h. Because of its elemental identity with the therapeutic agent ^212^Pb, it is suitable for the determination of image-derived individualized normal organ and tumor dosimetry-based treatment plans [3]. ^212^Pb (t_1/2_ = 10.6 h) itself decays via beta decay to ^212^Bi, which further decays via 36% alpha decay (6.1 MeV) to ^208^Tl and 64% beta decay to ^212^Po, followed by 100% alpha decay (8.8 MeV) to ^208^Pb. However, the stable ^208^Pb is in such low concentration, that no pharmacological side effects can occur (tracer principle). In addition to alpha and beta particles, there are several gamma and X-ray emissions, enabling post-therapeutic SPECT/CT imaging [4, 5]. The development of a novel Pb-specific chelator (PSC), together with a polyethylene glycol (PEG) linker has created a drug conjugate that promises improved tumor targeting, receptor binding, tumor accumulation and retention, along with a faster renal clearance than a DOTA counterpart [3, 6]. Further, the PSC-PEG_2_-unit has a higher ability to retain ^212^Bi after beta decay of complexed ^212^Pb compared to other chelators [7]. Preclinical evaluations have shown that [^212^Pb]Pb-VMT-α-NET therapies are effective in slowing tumor growth and resulted in complete durable responses that significantly improved survival rates in animal models compared to β-PRRT [6]. For example, fractionated injection regimens of ^212^Pb-labeled peptides have demonstrated tolerable safety profiles and effective tumor control, which are promising indicators for human clinical trials [8, 9]. Therefore, our goal was to perform individual theranostics with this radionuclide pair in a routine clinical setting with special emphasis of imaging performance, biokinetics, safety and efficiency for patients, which were resistant to standard PRRT and were no longer candidates for ^225^Ac-TAT.

## Materials and methods

### Patients

In this retrospective study, we analyzed data from patients who underwent imaging with [^203^Pb]Pb-VMT-α-NET and subsequent therapy with [^212^Pb]Pb-VMT-α-NET as a single-dose regimen between 03/2023 and 12/2023. Eligibility for lead-based theranostics included patients with a diagnosis of unresectable, metastatic neuroendocrine tumor with progressive disease after standard therapies, including somatostatin receptor analogues, chemotherapy, multiple cycles of PRRT with [^177^Lu]Lu-DOTATATE, [^225^Ac]Ac-DOTATATE, and radioembolization, if appropriate. Progression was defined by new SSTR-positive tumor lesions on [^68^ Ga]Ga-DOTATATE PET/CT imaging or by increasing blood tumor markers, namely Chromogranin A (CgA). Relatively preserved renal function (defined as eGFR > 30) and bone marrow function (defined as hemoglobin > 5 mmol/l, leukocytes within normal range, platelets > 100 GPt/l), was required. An Eastern Cooperative Oncology Group (ECOG) performance status of 0 to 2 was mandatory. Exclusion criteria were inadequate bone marrow reserve, urinary tract obstruction diagnosed with renal scintigraphy, missing clinical or imaging records and less than 3 months or missing of follow-up, including clinical assessment, blood sampling, renal scintigraphy and [^68^ Ga]Ga-DOTATATE PET/CT imaging. All patients were individually selected for VMT-α-NET theranostics by two senior nuclear medicine physicians with more than 10 years of experience in the field. All patients gave informed consent for imaging and treatment. The retrospective data analysis was approved by the local institutional ethics committee (ID number: BO-EK- 331082024). Clinical, demographic, and technical data were collected after inclusion in the study.

### Radiopharmaceutical preparation

Radiolabeling of PSC conjugates was performed according to standard protocols for these chelators. In brief, 0.1 µg/MBq of the precursor VMT-α-NET (PSC-PEG_2_-TOC, M = 1578.7 g/mol)) in H_2_O_suprapure_ was added to a 10 mL reaction vial together with 100 µL EtOH_absolute_, 290 µL 1 M NaAc/AcOH buffer (pH 4, 99,99% trace metal) and 2 mg sodium ascorbate (Ph.Eur.).

^203/212^Pb in 5–10 mL 1.6 M HCl_suprapure_ was trapped on custom-made Pb resin cartridge (100 mg PB-B10-F, Triskem, Bruz, France) preconditioned with 1 mL 2 M HCl_suprapure_. The trapped activity was rinsed with 1 mL 2 M HCl_suprapure_. The activity was eluted with 2 mL NaAc/AcOH buffer (pH 6, 99.99% trace metal) directly into the reaction vial. The solution was heated at 95 °C for 15 min for ^203^Pb and 15 or 45 min for ^212^Pb. Afterwards, the reaction solution was diluted and cooled with 4 mL 0.9% NaCl solution.

Following the labeling reaction, the product was purified using a C18 Plus light cartridge (WAT023501, Waters) preconditioned with 1 mL EtOH and 3 mL H_2_O (wet condition). The cooled and diluted product solution (4 mL 0.9% NaCl) was slowly passed over the C18 cartridge. The C18 cartridge containing the product was rinsed with 2 mL 0.9% NaCl solution and was directly eluted with 1 mL 50% EtOH for injection through a vented sterile filter (SLGVV255 F, Millex-GV, Merck) into a product vial. Finally, the product was diluted with 7 mL 0.9% NaCl solution. The radiochemical yield for [^203^Pb]Pb-VMT-α-NET was > 95% and for [^212^Pb]Pb-VMT-α-NET > 90%. Both radiotracers had radiochemical purities > 95% and were stable in final solution up to 48 h. Serum stabilities are reported by Li et al. [6].

One patient was administered [^212^Pb]Pb-VMT-α-NET that was produced using the same basic procedure, but adapted for an automated “Lead-it-EAZY” process, which is described elsewhere [10].

### Imaging with [^203^Pb]Pb-VMT-α-NET

Patients received a single intravenous bolus injection of [^203^Pb]Pb-VMT-α-NET (body weight adjusted activity 4.9 MBq/kg, mean activity 365 MBq, range 256–517 MBq). Whole-body scintigraphy and SPECT/CT acquisitions were conducted 10 min, 2, 10, 24 and, in some cases, up to 41 h after injection on a Symbia Intevo 6 SPECT/CT scanner (Siemens Healthineers) using medium-energy collimators. Images were obtained using an energy window at 279 keV (20% width) with adjacent upper and lower scatter windows (10% width). Whole-body scan speed was 8 cm/min and SPECT/CT scans were acquired with 120 projections (60 per detector, 30 s per projection) over a non-circular 360° orbit.

Regions of interest (ROI) for lesions and kidneys were drawn on SPECT/CT images to evaluate tumor and kidney uptake over time. Tumor-to-kidney ratios (T/K) per time point were calculated.

All patients underwent a [^99 m^Tc]Tc-MAG3 renal scintigraphy prior to injection of [^203^Pb]Pb-VMT-α-NET. Standardized blood tests were performed before and 24 h after injection. Patients underwent resting electrocardiograms before, 4 and 20 h p.i. Vital signs (pulse rate, blood pressure and body temperature) and potential adverse events were monitored up to 24 h p.i., the latter according to the Common Terminology Criteria for Adverse Events (CTCAE, [11]).

### Imaging and treatment with [^212^Pb]Pb-VMT-α-NET

The mean interval between the administration of [^203^Pb]Pb-VMT-α-NET and [^212^Pb]Pb-VMT-α-NET was 42 days (range 20–76 days).

Patients received a single slow intravenous infusion of [^212^Pb]Pb-VMT-α-NET at 1.2 MBq/kg (body weight adjusted, mean activity 100 MBq, range 86–109 MBq) over 15 min. Whole-body scans and SPECT/CT images were obtained 10 min, 2, 4 and 20 h p.i. with the same camera system as described above, equipped with high-energy collimators, using an energy window at 79 keV (40% width) with two adjacent scatter windows of 20% width. Whole-body imaging speed was 8 cm/min, and SPECT/CT scans were acquired with 120 projections (60 per detector, 30 s per projection) over a non-circular 360° orbit.

Prior to treatment, patients underwent a clinical evaluation, completed quality of life questionnaires (EORTC QLQ-C30 and EORTC QLQ-GINET21), underwent resting electrocardiography, [^99 m^Tc]Tc-MAG3 renal scintigraphy, and blood sampling. Patients received electrolyte infusion and standardized amino acid solution, containing arginine, cysteine and lysine for renal protection, at a flow rate of 150 mL/h for 10 h from 2 h before to 24 h after therapy. They were also treated with granisetron for antiemetic prophylaxis. Blood sampling, further examinations and patient monitoring were performed up to 48 h p.i. in the same manner as for [^203^Pb]Pb-VMT-α-NET imaging.

### Patient follow-up

All patients treated with [^212^Pb]Pb-VMT-α-NET underwent a clinical follow-up for 3 months after radiotracer injection. Blood samples and quality of life (QOL) questionnaires were taken every week for the first month after therapy and every two weeks thereafter. At 4 and 12 weeks after treatment, [^99 m^Tc]Tc-MAG3 renal scintigraphy and [^68^ Ga]Ga-DOTATATE PET/CT imaging were performed. Response assessment was determined by CgA measurements and metabolic and radiographic evaluation of PET/CT imaging. PET data were evaluated visually and quantitatively by two experienced nuclear medicine physicians (CB, EM) and compared to baseline scans. Metabolic tumor volume (MTV) was calculated from [^68^ Ga]Ga-DOTATATE PET scans at baseline and follow-up using MIM Software (version 7.3.6) with an SUV threshold of 2.5. Radiographic assessment was performed according to RECIST 1.1 criteria. Any increase in CgA and/or radiologic and/or metabolic evidence of new or growing tumor lesions was considered progressive disease. Adverse events were documented according to the CTCAE.

### Statistical analysis

Statistical analysis was performed with SPSS version 29.0 (IBM). The Student’s t-test was conducted to compare the means of normally distributed data and to derive p-values. Chi-squared test was used for non-normally distributed data. Changes in MTV were validated using the Wilcoxon signed-rank test. Cohen’s effect size was used to interpret changes in QLQ-C30 scores. Significance was set at *p* < 0.05 (two-tailed).

## Results

### [^203^Pb]Pb-VMT-α-NET

Twelve patients (3 female, 9 male) with unresectable, metastatic, progressive NET of various origins and grades underwent [^203^Pb]Pb-VMT-α-NET whole body scintigraphy and SPECT/CT imaging (see Table 1 for patient characteristics). All patients had an ECOG performance status of 0–2.

**Table 1:** Patient characteristics

Patient no	Age	Sex	Type of NET	Tumor grade	Ki- 67 (%)	Chromo-granin A (ng/ml)	GFR (mL/min/1,73)	Hemoglobin (mmol/L)	WBC (GPt/L)	Platelets (GPt/L)	Lympho-cytes (GPt/L)	Time since diagnosis (months)	Pretreatment courses
1	75	W	CUP	3	34	2938.0	31	6.3	9.40	160	1.22	113	SSA, Everolimus, Temozolomid/Capecitabine, 4.7 GBq 177-Lu-DOTATATE, 10.4 MBq Ac- 225-DOTATATE, Radioembolization
2	80	M	Small bowel	3	30	3321.0	81	7.7	5.01	234	0.68	65	Everolimus/Sunitinib, 36.5 GBq Lu- 177-DOTATATE
3	65	M	CUP	NA	NA	34.9	78	8.3	5.37	263	1.18	40	SSA, 7.4 GBq 177-Lu-DOTATATE, 10.6 MBq Ac- 225-DOTATATE
4	72	M	Small bowel	2	3	1713.0	90	6.6	3.91	303	0.37	55	SSA, Everolimus, 21.2 GBq Lu- 177-DOTATATE, 4.3 MBq Ac- 225-DOTATATE
5	82	M	Bronchial Carcinoid	2	15	1550.0	62	8.3	8.29	198	2.50	11	Denosumab, 10.9 GBq Lu- 177-DOTATATE
6	65	W	Pancreatic	3	35	75.6	88	7.3	8.78	263	0.61	34	Everolimus, Streptozocin/ 5-FU, 26.9 GBq Lu- 177-DOTATATE, 3.9 MBq Ac- 225-DOTATATE
7	71	M	Pancreatic	3	> 20	4800.0	67	8.4	13.09	253	1.61	80	SSA, Denosumab, 18.6 GBq Lu- 177-DOTATATE
8	63	M	CUP	2	18	3317.0	67	8.4	10.17	310	1.85	36	SSA, 20.5 GBq Lu- 177-DOTATATE
9	60	M	Rectal	1	< 2	23.8	90	7.7	3.32	241	0.51	47	SSA, Denosumab, 15.8 GBq Lu- 177-DOTATATE, 7.7 MBq Ac- 225-DOTATATE
10	71	W	Small bowel	1	< 2	6661.0	45	7.0	6.17	307	0.76	49	SSA, FOLFOX, 6.1 GBq Lu- 177-DOTATATE, 4.9 MBq Ac- 225-DOTATATE
11	84	M	Small bowel	2	5	1927.0	73	8.9	3.38	137	1.02	53	18.6 GBq Lu- 177-DOTATATE
12	65	M	Pancreatic	2	5	2983.0	57	5.6	4.03	371	0.54	104	Streptozotocin/Doxorubicin, 22.3 GBq Lu- 177-DOTATATE

*CUP* cancer of unknown primary, *SSA* somatostatin analogues, *NA* not available

**Fig. 2:**
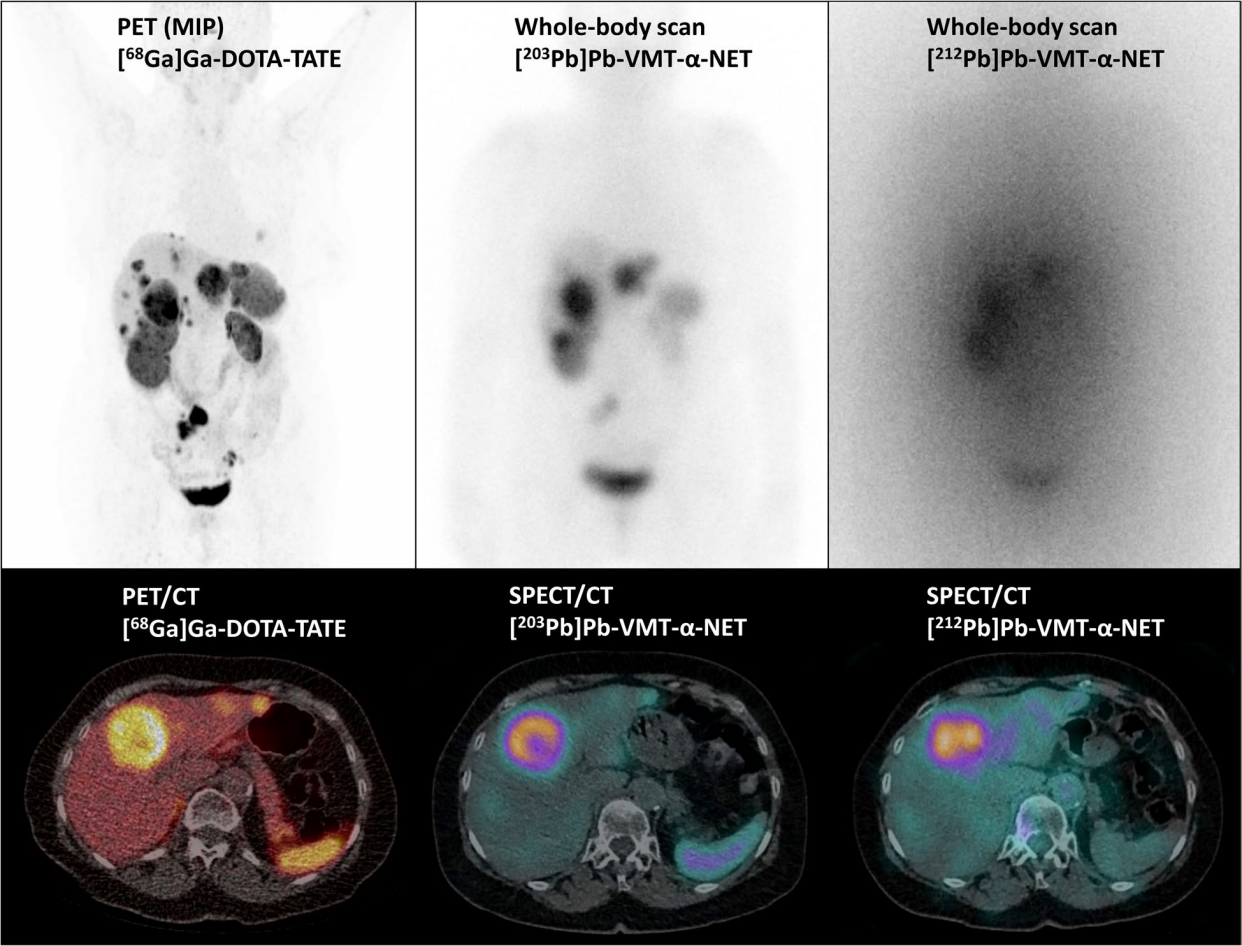
Transversal slice and maximum intensity projection (MIP) of [^68^ Ga]Ga-DOTATATE PET/CT imaging of Patient No. 9 with GEP NET G1 (left column). Whole body scans and corresponding transversal slices of pre-treatment imaging 2.5 h post-injection of 243 MBq [^203^Pb]Pb-VMT-α-NET (center column) and post-treatment imaging at 2.5 h after administration of 86 MBq [^212^Pb]Pb-VMT-α-NET (right column). The scintigraphic images showed a high accumulation of [^203/212^Pb]Pb-VMT-α-NET in liver metastases, comparable to the [^68^ Ga]Ga-DOTATATE PET/CT images

**Fig. 3:**
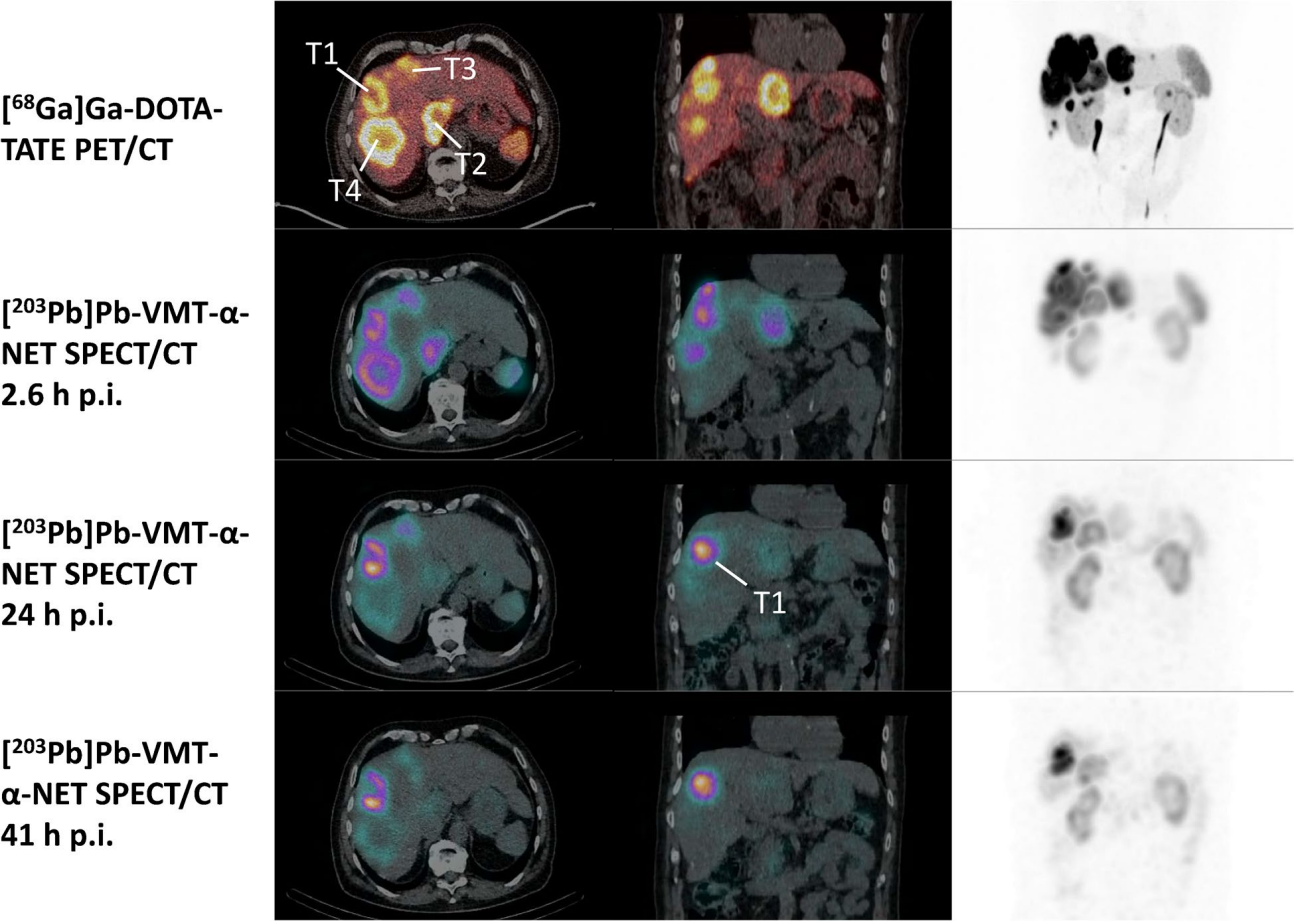
First row: Transversal and coronal slices of [^68^ Ga]Ga-DOTATATE PET/CT fusion images and maximum intensity projections (MIP) of Patient No. 2 with GEP NET G3 and multiple liver metastases before administration of [^203^Pb]Pb-VMT-α-NET. Four tumor lesions (T1-T4) were identified for dosimetry. Second to fourth row: Transversal and coronal slices of SPECT/CT images and MIP of the same patient at 2.6, 24 and 41 h of after administration of 348 MBq [^203^Pb]Pb-VMT-α-NET. Note the initial intense uptake in all tumor lesions at 2.6 h p.i. and the changing tracer accumulation in all 4 lesions over time

**Fig. 4:**
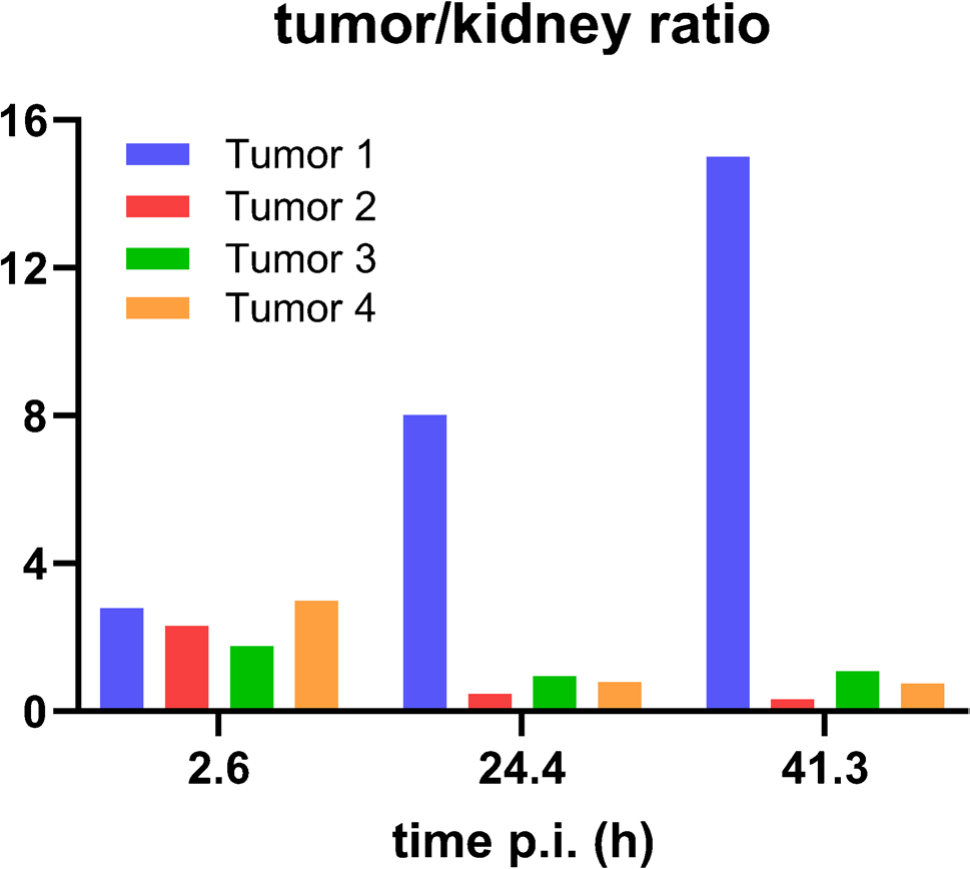
Tumor/kidney ratios of four liver tumor lesions over time (see Fig. 2), obtained on SPECT/CT images after administration of [^203^Pb]Pb-VMT-α-NET. Note the continuously increasing ratio in Tumor 1 and the initially decreasing ratio in the other lesions, which remains stable after 24 h

After the administration of the radiotracer, all tumor lesions demonstrated high uptake levels, which were markedly higher than those in the liver and renal parenchyma, up to 3 h post-injection. Up to this time point, visual characterization showed a relatively comparable tumor and normal tissue uptake to [^68^ Ga]Ga-DOTATATE PET (Fig. 2). However, we observed relevant differences in tumor activity accumulation over time: visual and initial biokinetic data revealed inter- and intra-individually heterogenous tumor uptake that was either sustained, increasing or decreasing at all time points assessed (Fig. 3). Correspondingly, tumor-to-kidney (T/K) ratios ranged from 0.5 to 15.0 between tumors in the same patient, and also for the same lesion between 3 and 41 h (Fig. 4).

Two patients (patient No. 5 and 6) showed low overall tumor uptake on [^203^Pb]Pb-VMT-α-NET SPECT/CT imaging (below normal liver parenchyma levels), which appeared to be in contrast with moderate tumor uptake levels on [^68^ Ga]Ga-DOTATATE PET imaging, and were therefore not eligible for [^212^Pb]Pb-VMT-α-NET therapy.

The administration of [^203^Pb]Pb-VMT-α-NET was uncomplicated and well tolerated in all patients. There were no significant changes in vital signs or corrected QT interval. No hematologic side effects were observed. Renal and hepatic function parameters remained unchanged. Two patients (16.6%) experienced mild nausea (CTCAE grade 1), but no vomiting. Two serious adverse events were reported, namely two deaths (patient No. 4 and 7) due to disease progression. Both events were not related to the administration of [^203^Pb]Pb-VMT-α-NET. No other adverse events were reported.

### [^212^Pb]Pb-VMT-α-NET

**Fig. 5:**
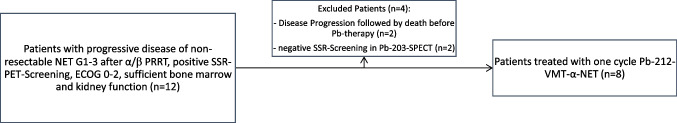
Study flow chart

8 of 12 patients (2 female, 6 male, see Table 1: patient No. 1–3 and 8–12) were treated with one cycle of [^212^Pb]Pb-VMT-α-NET (study flow chart: Fig. 5). Two patients (patient No. 4 and 7) died before treatment due to disease progression. As mentioned above, another two patients (patient No. 5 and 6) showed poor tumor uptake on [^203^Pb]Pb-VMT-α-NET SPECT/CT imaging, making them ineligible for TAT with [^212^Pb]Pb-VMT-α-NET.

Post-therapeutic SPECT/CT imaging showed visually relevant, but lesion- and time-point-dependent heterogeneous tumor and normal tissue uptake, similar to the findings of [^203^Pb]Pb-VMT-α-NET-imaging (Fig. 2).

No high grade or severe side effects were evident (CTCAE 4–5). A total of 23 adverse events were reported. 2 of the 23 events (8.7%) were grade 3, i.e. nausea and vomiting on the day of treatment, all of which were effectively treated with antiemetic medication and resolved within 24 h. Another 8 (34.7%) events were grade 2, including 6 nausea without vomiting and 2 fatigue. Six patients reported grade 1–2 fatigue for 2–4 weeks after treatment. One patient (No. 10) noted a slight decline in general condition at 3 months (ECOG 1 to ECOG 2). Based on the stable disease status confirmed by [^68^ Ga]Ga-DOTATATE PET and CgA levels, this adverse event was considered to be related to the administration of [^212^Pb]Pb-VMT-α-NET and not to disease progression.

There were no clinically relevant changes in vital signs or corrected QT interval in any of the patients.

Regarding renal function, the mean baseline eGFR for all subjects was 65 mL/min/1.73 m^2^. Twenty-four hours post-treatment, mean eGFR was 64 mL/min/1.73 m^2^, and at 3 months post-treatment, eGFR was 63 mL/min/1.73 m^2^, which was not significantly different from baseline (*p* = 0.31). Renal sequence scintigraphy was unchanged in all cases with respect to side separated kidney function. Hemoglobin levels remained unaltered during follow-up (7.5 mmol/L vs. 7.4 mmol/L at baseline; *p* = 0.734), as did absolute platelet counts (250.0 GPt/L vs. 260.6 GPt/L, *p* = 0.263) and absolute leukocyte counts (5.19 GPt/L vs. 6.53 GPt/L, *p* = 0.441). However, a statistically significant decrease in absolute lymphocyte count was observed at 3 months (0.855 GPt/L vs. 0.939 GPt/L at baseline, *p* = 0.035). The lymphocyte count reduction could be observed in 5 of 8 patients, while the other 3 patients showed stable lymphocyte counts. Liver function parameters such as transaminases and inflammatory parameters such as CRP remained unchanged.

**Fig. 6:**
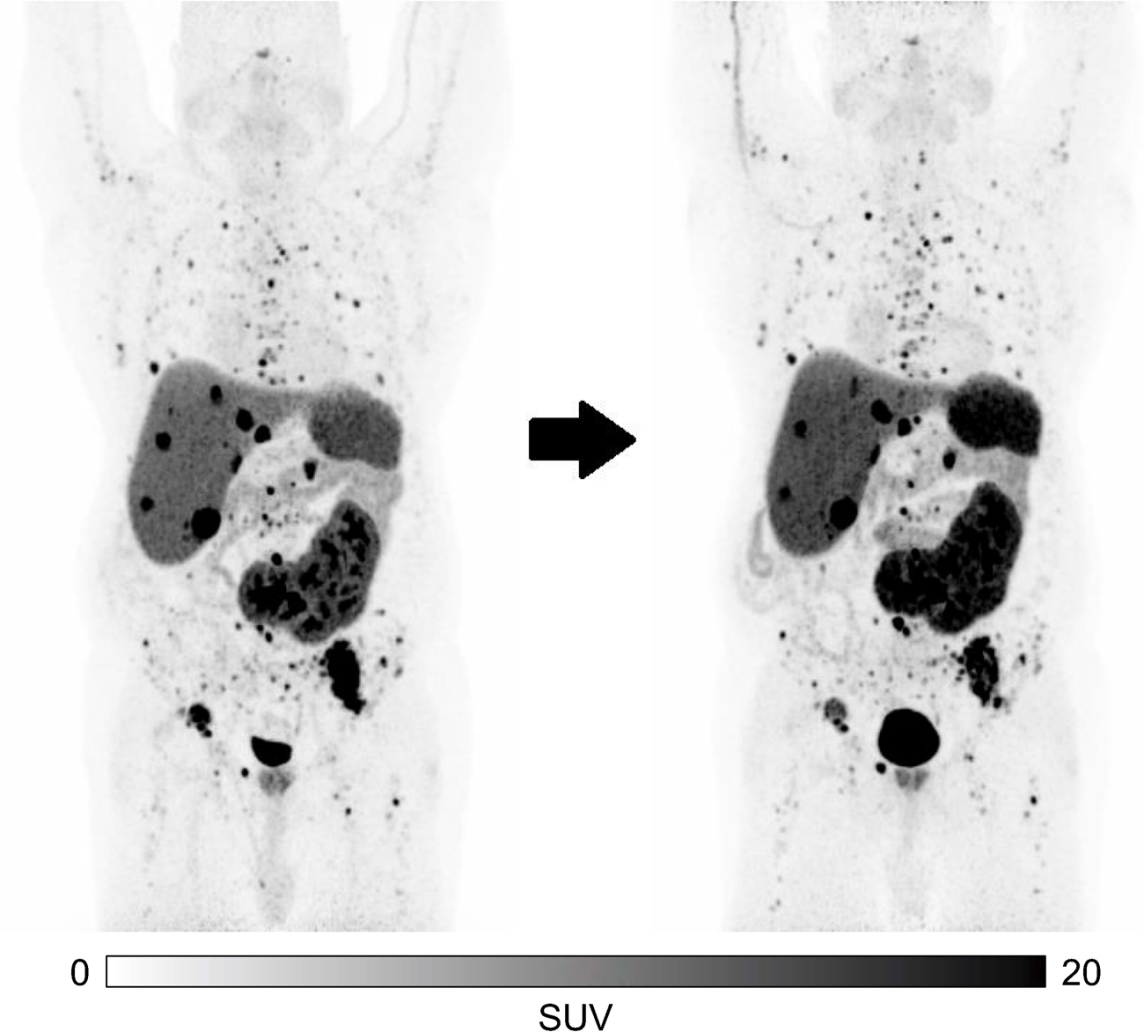
[^68^ Ga]Ga-DOTATATE PET/CT maximum intensity projections (MIP) of patient No. 8 with G2-CUP NET, multiple hepatic, osseous and lymphonodal metastases at baseline (left) and follow-up (right) 3 months after administration of 108 MBq [^212^Pb]Pb-VMT-α-NET. Tumor burden, as well as tumor marker CgA, remained stable over time (3246 vs. 3317 ng/ml at baseline)

**Fig. 7:**
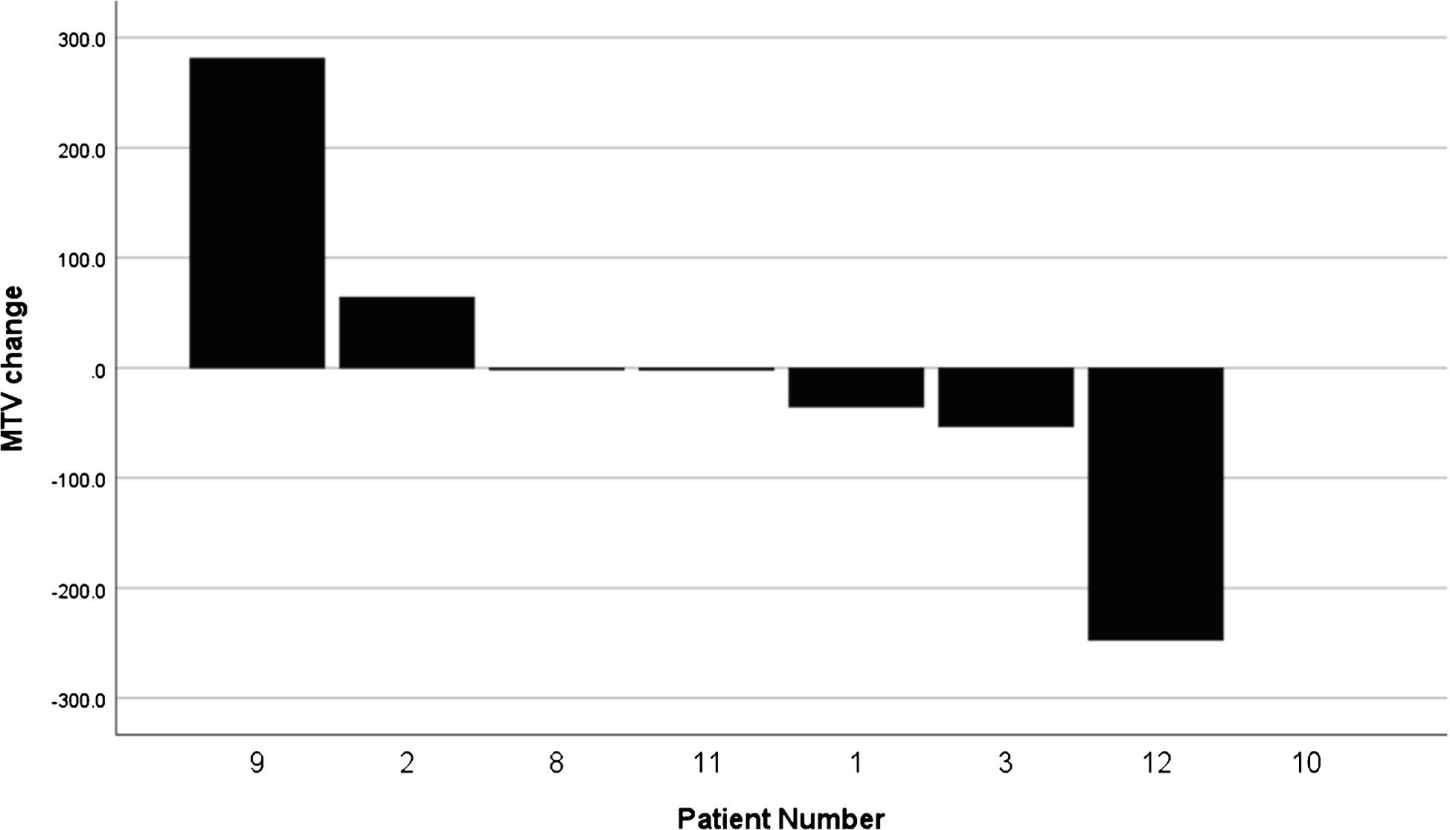
Waterfall plot of the change in metabolic tumor volume (in cm^3^) after 1 cycle of therapy with [^212^Pb]Pb-VMT-α-NET

Response assessment by [^68^ Ga]Ga-DOTATATEPET/CT at 1 month demonstrated stable disease in 7 of 8 patients (88%). One patient (patient No. 8) showed an increasing SUVmax in most tumor lesions and better delineation of multiple osseous lesions that were not clearly visible at baseline PET – this was considered a flare phenomenon, as the control PET 3 months after therapy showed a stable tumor burden without any new lesions (Fig. 6). Overall, all patients (100%) showed stable disease on [^68^ Ga]Ga-DOTATATE PET/CT 3 months after one cycle of Pb- 212-VMT-α-NET, according to RECIST criteria, visual and quantitative metabolic assessment. Mean metabolic tumor volume at baseline was 392.6 cm^3^ (range 72.2–1120.2 cm^3^) vs. 393.3 cm^3^ at follow-up (range 70.6–1401.3 cm^3^, *p* = 0.735). Absolute changes in MTV are shown in Fig. 7. The MTV of patient No. 10 could not be evaluated due to inconsistent raw data that could not be processed by the MIM software. The tumor marker CgA decreased significantly at 3 months (1424.3 vs. 2650.7 ng/ml, *p* = 0.05). Half of the patients showed a relevant decrease in CgA, with a maximum decline from 6661.0 to 1300.0 ng/ml (patient No. 10), while the other half showed stable tumor markers. Interestingly, the tumor marker reduction did not translate to PET/CT imaging. All subjects in the treatment group were alive at the time of the last data collection (up to 8 months p.i.).

Evaluation of the EORTC questionnaires revealed no significant changes in quality of life or functional dimensions.

## Discussion

This study provides first clinical insights into the treatment of heavily pretreated patients with [^212^Pb]Pb-VMT-α-NET, together with pre-therapeutic assessment with [^203^Pb]Pb-VMT-α-NET SPECT/CT. Subjects treated with lead-based TAT showed an intense intratumoral signal on [^203^Pb]Pb-VMT-α-NET SPECT/CT, indicating high SSTR2 receptor affinity. All patients demonstrated stable disease up to 8 months after only one cycle of therapy, which is remarkable considering the conservative dose regimen administered, the long treatment history of most patients, and the mostly high tumor burden and grade, with 10 of 12 patients suffering from metastatic grade 2 or 3 NET (83%).

It is certainly too early to accurately compare the efficacy of [^212^Pb]Pb-VMT-α-NET with other alpha emitters in the field, especially since the current study was not designed to do so. Theoretically, the so-called in vivo alpha particle generator ^212^Pb offers fewer opportunities to damage surrounding healthy tissue than ^225^Ac due to its single alpha emission and fewer daughter nuclides in its decay chain [12]. Together with its short half-life of only 10.6 h vs. 9.92 days, resulting in higher dose rates, and the lower recoil energy for lower detachment rates from the target molecule, ^212^Pb should be as at least comparably effective to ^225^Ac, with a lower rate of potential side effects [8, 13]. Another future argument for lead-based TAT may be the more flexible logistics on a daily basis, as ^212^Pb is a generator product, with ^224^Ra or ^228^Th as the parent nuclide [14].

Currently, there is no recommendation available regarding the administered dose per cycle. A phase I/IIa clinical trial aiming to establish a recommended dose regime is underway [15]. Nevertheless, it should be noted that patients pretreated with systemic PRRT-based therapies are excluded from this study, which may have a serious impact on the maximum tolerated dose and may be of limited significance to real-world clinical populations. An Indian study group administered 2.50 MBq/kg/cycle [^212^Pb]Pb-VMT-α-NET for several cycles every 8 weeks in untreated and pretreated NET patients, with a resulting toxicity read-out suggesting the potential for further dose escalation [16]. Although lacking comparability, Delpassand et al. proposed a dose regimen of 2.50 MBq/kg/cycle [^212^Pb]Pb-DOTAMTATE, a bifunctional metal chelator also targeting SSTR [17]. The tumor and normal organ dosimetry is still pending and will be published in a subsequent manuscript. However, since we did not observe any serious side effects under our conservative dose regimen, it may be possible to further escalate the administered doses up to 2.50 MBq/kg or beyond, although long-term efficacy and toxicity data are needed to prove this proposition.

In the current study, the safety profile of both [^203/212^Pb]Pb-VMT-α-NET was promising. No therapeutically relevant side effects were observed with the administration of [^203^Pb]Pb-VMT-α-NET. The mean activity administered for SPECT imaging was higher than in other observational studies published to date (365 MBq vs. 275 MBq [18] vs. 260 MBq [19]). We proactively used higher activities to account for the relatively low count ratesreported by Dos Santos et al. [18] and to enable SPECT/CT imaging with tolerable acquisition times. In the search for the optimal imaging time point, we investigated several time windows, ranging from scanning 10 min after injection, through 2 and 10 h p.i. up to 24 h p.i. By visual approach, the best imaging quality was seen around 2 h after injection. This time window is roughly supported by first results of a phase 0 study by Jain et al. [20], who suggest best imaging time points at 4 h post injection, closely followed by imaging at 1 h p.i.

With regard to uptake and biokinetic evaluation with [^203^Pb]Pb-VMT-α-NET SPECT/CT, we surprisingly observed a high variability of tumor activity accumulation both intra- and inter-tumoral over time, with the best agreement with [^68^ Ga]Ga-DOTATATE PET imaging in the first 3 h p.i. (as mentioned above). This observation was not related to tumor grade, tumor burden, pretreatment course, or blood tumor marker levels. However, these results are partially comparable to in vivo data from AR42 J xenograft mice [3], which also showed increasing but not decreasing T/K ratios over time for single tumor lesions. In our opinion, this could be related to an individual tumor biology (as a result of different stages of dedifferentiation), which we can neither prove nor rule out due to the lack of histological samples. Further work to truly understand the whole-body biokinetics of these radiotracers is ongoing, with determination of time-integrated activity coefficients for both tumor and normal tissues, with the ultimate goal of achieving the possibility of individual predictive dose estimation for therapy with [^212^Pb]Pb-VMT-α-NET.

TAT with [^212^Pb]Pb-VMT-α-NET was well tolerated by all patients treated, with the typical PRRT side effects of nausea, vomiting and fatigue, which can be attributed to the amino acid solution administered. As expected, there were no changes in vital signs or ECG parameters were seen. Renal function was unaltered during treatment and follow-up. Most hematologic parameters remained unchanged, but the absolute lymphocyte count decreased significantly up to three months after therapy. This effect was also observed in the dose-escalation study of Delpassand et al. [17], with no long-term clinical consequences reported to date. No hepatic toxicity was noted.

To date, this is the first in-human study of a real-world population comprising heavily pretreated patients with various metastatic NETs receiving lead-based TAT.

Limitations of this study include its single-center, retrospective design and relatively small cohort. All study patients had received intensive multimodality treatment prior to TAT with VMT-α-NET in a compassionate setting and were therefore carefully selected. The results of this study concerning efficiency and toxicity should be treated with caution, as only one cycle of lead-based TAT was performed and the follow-up period was limited. While the toxicity profile of [^203^Pb]Pb-VMT-α-NET can be considered benign, the long-term effects of [^212^Pb]Pb-VMT-α-NET are still unknown, especially after multiple cycles with higher dose regimens. Because of the half-life of ^212^Pb, these multiple cycles might be performed in shorter periods compared to ^90^Y, ^177^Lu or ^225^Ac, considering the patient’s condition. Another limitation is posed by the protracted mean interval of 42 days between imaging with ^203^Pb and therapy with ^212^Pb, possibly resulting in a biological alteration in tumor masses and consequently modify therapy outcomes. This duration exceeds that of a standard PRRT with [^177^Lu]Lu-DOTATATE, due to the current limited availability of ^203/212^Pb-radiotracers and the concomitant logistical challenges. In order to address this, we compared the CgA biomarker levels at the time of imaging and therapy. This revealed a tendency for rising CgA prior to therapy, although this rise did not reach statistical significance (2387.7 vs. 2650.7 ng/ml, *p* = 0.055). Given that we performed assessment of therapy efficacy with [^68^ Ga]Ga-DOTATATE PET/CT, the encouraging results of our single-cycle lead-based TAT are therefore not diminished in any way. In subsequent studies, the time interval between ^203^ and ^212^Pb should be reduced through optimization of planning and logistics to ensure enhanced comparability of these theranostic agents.

Ultimately, prospective analyses with a larger number of patients are needed to further elucidate the findings of the recent work, with special attention to the primary goal of establishing personalized dosimetry. This includes, for example, qualitative and quantitative monitoring response to therapy using [^203/212^Pb]Pb-VMT-α-NET SPECT/CT, which has the potential to supersede [^68^ Ga]Ga-DOTATATE-PET/CT for this purpose. As another important step towards standardization of the theranostic process, a GMP-compliant automated synthesis has been developed to enable other nuclear medicine institutions to produce [^212^Pb]Pb-VMT-α-NET by a harmonized method [21].

## Conclusion

Imaging with [^203^Pb]Pb-VMT-α-NET followed by PRRT with [^212^Pb]Pb-VMT-α-NET is well tolerated and safe to perform according to initial experience, with encouraging efficacy even in a heterogeneous and heavily pretreated patient population. All patients demonstrated stable disease up to 8 months after only one cycle of therapy, which is remarkable considering the conservative dose regimen administered, the long treatment history of most patients, and the mostly high tumor burden and grade, with 10 of 12 patients suffering from metastatic grade 2 or 3 NET (83%). Future studies will evaluate the potential of [^203^Pb]Pb-VMT-α-NET SPECT/CT in terms of patient selection and individualized dosimetry for TAT. Furthermore, additional research is required to assess the tolerability and efficacy of [^212^Pb]Pb-VMT-α-NET (at higher administered levels of radioactivity) over multiple treatment cycles in larger subject cohorts.

## Data Availability

The data sets generated during and/or analyzed during the current study are available from the corresponding author on reasonable request.

## Abbreviations

NET: Neuroendocrine Tumor
PRRT: Peptide receptor radionuclide therapy
SSTR2: Somatostatin subtype 2 receptor
TAT: Targeted alpha therapy
VMT: Viewpoint Molecular Targeting
PSC: Pb-specific chelator
PEG: Polyethylene glycol
CgA: Chromogranin A
eGFR: Estimated glomerular filtration rate
SUV: Standardized uptake value
ROI: Region of interest
CTCAE: Common Terminology Criteria for Adverse Events
EORTC QLQ: European Organisation for Research and Treatment of Cancer Quality of life questionnaire
QOL: Quality of life
MTV: Metabolic tumor volume
RECIST: Response Evaluation Criteria in Solid Tumors
ECOG: Eastern Cooperative Oncology Group
RECIST: Response Evaluation Criteria In Solid Tumors
MIP: Maximum intensity projection

## References

[1] Gape PMD, Schultz MK, Stasiuk GJ, Terry SYA. Towards effective targeted alpha therapy for neuroendocrine tumours: a review. Pharmaceuticals (Basel). 2024;17(3):334. 10.3390/ph17030334. 38543120 10.3390/ph17030334 PMC10974115

[2] Hennrich U, Kopka K. Lutathera®: the first FDA- and EMA-approved radiopharmaceutical for peptide receptor radionuclide therapy. Pharmaceuticals (Basel). 2019;12(3):114. 10.3390/ph12030114. 31362406 10.3390/ph12030114 PMC6789871

[3] Lee D, Li M, Liu D, Baumhover NJ, Sagastume EA, Marks BM, et al. Structural modifications toward improved lead-203/lead-212 peptide-based image-guided alpha-particle radiopharmaceutical therapies for neuroendocrine tumors. Eur J Nucl Med Mol Imaging. 2024;51(4):1147–62. 10.1007/s00259-023-06494-9. 37955792 10.1007/s00259-023-06494-9 PMC10881741

[4] Michler E, Kästner D, Brogsitter C, Pretze M, Hartmann H, Freudenberg R, et al. First-in-human SPECT/CT imaging of [212Pb]Pb-VMT-α-NET in a patient with metastatic neuroendocrine tumor. Eur J Nucl Med Mol Imaging. 2024;51(5):1490–2. 10.1007/s00259-023-06529-1. 37991526 10.1007/s00259-023-06529-1 PMC10957691

[5] Kästner D, Michler E, Hartmann H, Pretze M, Freudenberg R, Schultz MK, et al. PP3106 203/212Pb theranostic isotopes for targeted alphatherapy – SPECT imaging properties and first clinical experience. Phys Med. 2024;125(Supplement 1):103873. 10.1016/j.ejmp.2024.103873.

[6] Li M, Baumhover NJ, Liu D, Cagle BS, Boschetti F, Paulin G, et al. Preclinical evaluation of a lead specific chelator (PSC) conjugated to radiopeptides for 203Pb and 212Pb-based theranostics. Pharmaceutics. 2023;15(2):414. 10.3390/pharmaceutics15020414. 36839736 10.3390/pharmaceutics15020414 PMC9966725

[7] Bauer D, Carter LM, Atmane MI, De Gregorio R, Michel A, Kaminsky S, et al. 212Pb-pretargeted theranostics for pancreatic cancer. J Nucl Med. 2024;65(1):109–16. 10.2967/jnumed.123.266388. 37945380 10.2967/jnumed.123.266388 PMC10755526

[8] Miederer M, Benešová-Schäfer M, Mamat C, Kästner D, Pretze M, Michler E, et al. Alpha-emitting radionuclides: current status and future perspectives. Pharmaceuticals (Basel). 2024;17(1):76. 10.3390/ph17010076. 38256909 10.3390/ph17010076 PMC10821197

[9] Kokov KV, Egorova BV, German MN, Klabukov ID, Krasheninnikov ME, Larkin-Kondrov AA, et al. 212Pb: production approaches and targeted therapy applications. Pharmaceutics. 2022;14(1):189. 10.3390/pharmaceutics14010189. 35057083 10.3390/pharmaceutics14010189 PMC8777968

[10] Pretze M, Michler E, Kästner D, Kunkel F, Sagastume EA, Schultz MK, et al. Lead-it-EAZY! GMP-compliant production of [212Pb]Pb-PSC-PEG2-TOC. EJNMMI Radiopharm Chem. 2024;9(1). 10.1186/s41181-024-00305-8. 10.1186/s41181-024-00305-8 PMC11602913 39604560

[11] National Cancer Institute. Common terminology criteria for adverse events. Definitions. 2020.

[12] Sartor O, Sharma D. Radium and other alpha emitters in prostate cancer. Transl Androl Urol. 2018;7(3):436–44. 10.21037/tau.2018.02.07. 30050802 10.21037/tau.2018.02.07 PMC6043743

[13] Guérard F, Gestin JF, Brechbiel MW. Production of [(211)At]-astatinated radiopharmaceuticals and applications in targeted α-particle therapy. Cancer Biother Radiopharm. 2013;28(1):1–20. 10.1089/cbr.2012.1292. 23075373 10.1089/cbr.2012.1292 PMC3545490

[14] Jang A, Kendi AT, Johnson GB, Halfdanarson TR, Sartor O. Targeted alpha-particle therapy: a review of current trials. Int J Mol Sci. 2023;24(14):11626. 10.3390/ijms241411626. 37511386 10.3390/ijms241411626 PMC10380274

[15] Prasad V, Trikalinos N, Hanna A, Johnson F, Puhlmann M, Wahl R. A phase I/IIa of [212Pb]VMT-a-NET targeted alpha-particle therapy for advanced SSTR2 positive neuroendocrine tumors. J Nucl Med. 2024;65(supplement 2):242430–242430.

[16] Sen I, Malik D, Thakral P, Schultz M. 212Pb-VMT-Î±-NET targeted alpha therapy in metastatic neuroendocrine tumors: first in human study on safety and efficacy. J Nucl Med. 2024;65(supplement 2):242556–242556.

[17] Delpassand ES, Tworowska I, Esfandiari R, Torgue J, Hurt J, Shafie A, et al. Targeted α-emitter therapy with 212Pb-DOTAMTATE for the treatment of metastatic SSTR-expressing neuroendocrine tumors: first-in-humans dose-escalation clinical trial. J Nucl Med. 2022;63(9):1326–33. 10.2967/jnumed.121.263230. 34992153 10.2967/jnumed.121.263230 PMC9454455

[18] dos Santos JC, Schäfer M, Bauder-Wüst U, Lehnert W, Leotta K, Morgenstern A, et al. Development and dosimetry of 203Pb/212Pb-labelled PSMA ligands: bringing “the lead” into PSMA-targeted alpha therapy? Eur J Nucl Med Mol Imaging. 2019;46(5):1081–91. 10.1007/s00259-018-4220-z. 30603987 10.1007/s00259-018-4220-z PMC6451745

[19] Thakral P, Sen IB, Das SS, Schultz MK, Kumari J, Virupakshappa CB, et al. Lead-203 VMT-α-neuroendocrine tumor scintigraphy: a promising theranostics agent. Indian J Nucl Med. 2024;39(2):142. 10.4103/ijnm.ijnm_2_23. 38989305 10.4103/ijnm.ijnm_2_23 PMC11232724

[20] Jain S, Graves S, Bodeker K, Gaimari-Varner K, Chandrasekharan C, Schultz M, et al. Optimal imaging timepoint for diagnostic performance of 203Pb-VMT-α-NET SPECT/CT in neuroendocrine tumors. J Nucl Med. 2024;65(supplement 2):242347–242347.

[21] Pretze M, Michler E, Kästner D, Kunkel F, Sagastume EA, Schultz MK, et al. Lead-it-EAZY! GMP-compliant production of [212Pb]Pb-PSC-PEG2-TOC. EJNMMI Radiopharm Chem. 2024;9(1):81. 10.1186/s41181-024-00305-8. 39604560 10.1186/s41181-024-00305-8 PMC11602913

